# Translational control in cortical development

**DOI:** 10.3389/fnana.2022.1087949

**Published:** 2023-01-09

**Authors:** Federico Cremisi, Robert Vignali

**Affiliations:** ^1^Laboratory of Biology, Department of Sciences, Scuola Normale Superiore, Pisa, Italy; ^2^Department of Biology, University of Pisa, Pisa, Italy

**Keywords:** cortex, evolution, development, microRNA, RNA binding protein

## Abstract

Differentiation of specific neuronal types in the nervous system is worked out through a complex series of gene regulation events. Within the mammalian neocortex, the appropriate expression of key transcription factors allocates neurons to different cortical layers according to an inside-out model and endows them with specific properties. Precise timing is required to ensure the proper sequential appearance of key transcription factors that dictate the identity of neurons within the different cortical layers. Recent evidence suggests that aspects of this time-controlled regulation of gene products rely on post-transcriptional control, and point at micro-RNAs (miRs) and RNA-binding proteins as important players in cortical development. Being able to simultaneously target many different mRNAs, these players may be involved in controlling the global expression of gene products in progenitors and post-mitotic cells, in a gene expression framework where parallel to transcriptional gene regulation, a further level of control is provided to refine and coordinate the appearance of the final protein products. miRs and RNA-binding proteins (RBPs), by delaying protein appearance, may play heterochronic effects that have recently been shown to be relevant for the full differentiation of cortical neurons and for their projection abilities. Such heterochronies may be the base for evolutionary novelties that have enriched the spectrum of cortical cell types within the mammalian clade.

## Introduction

The mammalian isocortex is a complex telencephalic structure made of six cell layers, each harboring precise types of neurons with appropriate identities and connectivity. This structure is progressively built during development, from a set of neural progenitor cells (NPCs) within the pallium that generate, directly or indirectly, the six layers, following an inside-outside model. Layer I is the most external layer and the first to be generated, followed by deep (infra-granular) layer VI-V neurons projecting extra-cortically, then by granular layer IV neurons receiving thalamic afferents, and eventually by superficial (supra-granular) layer III-II neurons projecting intracortically (Cadwell et al., 2019). GABAergic inhibitory neurons are instead mostly generated by sub-pallial embryonic NPCs migrating dorsally (Jones, 2009).

**Figure 1:**
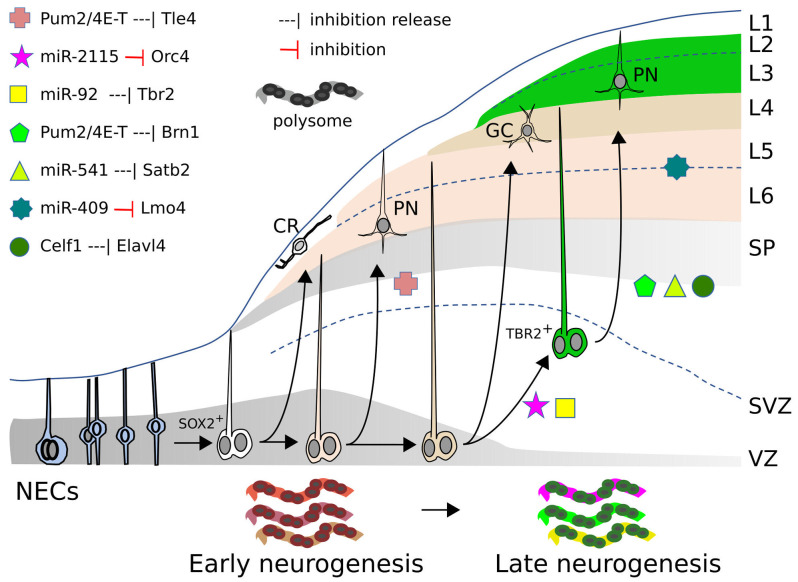
miRs, RBP, and RP action in mammalian neocortex. Neocorticogenesis is depicted from NECs to early and late corticogenesis, during the generation of cortical cell layers (L1–L6). The different symbols represent the inhibitory action, exerted by miRs or RBPs, on mRNAs of the indicated genes (or the release of this inhibition), that allow cell state transitions, for some examples detailed in the text. At the bottom, the dynamic ”ribosome signature” is also illustrated, with different colors symbolizing the changing repertoire of RPs and mRNAs recruited on polysomes. SOX2^+^: apical radial glia. TBR2^+^: basal/outer radial glia. CR, Cajal-Retzius cell; GC, granule cell; PN, pyramidal neuron; SP, subplate; SVZ, subventricular zone; VZ, ventricular zone.

Pallium and sub-pallium are shared by all vertebrates, with the sub-pallium much more conserved than the pallium also at the gene expression level (Woych et al., 2022). Single cell RNA sequencing allows to monitor the changing repertoire of transcripts expressed in cohorts of developing cortical cells as they transit from their initial state of neuroepithelial cells (NECs) to NPCs, with progressively more restricted differentiation potential as radial glial cells (RGs), basal/outer radial glial cells (bRG/oRGs) and intermediate progenitor cells (IPCs), to eventually become the post-mitotic neurons (PMNs) of the different layers (Cadwell et al., 2019; Figure 1). The final identity of a cortical neuron is achieved by the coordinated activation of a combination of master genes encoding key cortical transcription factors (CTFs), such as *Satb2, Bcl11b/Ctip2, Fezf2, Tbr1* (Kast and Levitt, 2019). The activation of distinct master genes occurs at specific times during the progressive restriction of the cell differentiative potential, which takes place during cortical layering. The combination of CTFs active in an NPC when it stops dividing accounts for the type of neuron it will become (e.g., granular, infra- or supra-granular neuron) and for its anterior-posterior or medial-lateral positional identity (Lodato and Arlotta, 2015). How such a genetic program evolved remains an open question, but a common repertoire of CTFs controlling pallial development has been amazingly conserved during vertebrate evolution (Woych et al., 2022).

### Cortical evolution

Few new master genes have been recruited during evolution for building the pallium. One example is *Satb2*, expressed in the dorsal pallium of sauropsids and mammals, but not of amphibians (Woych et al., 2022). Nonetheless, it seems that the interaction between master genes, rather than the enrollment of other genes, is pivotal for pallial evolution. For example, the sole interaction of *Satb2* with other pallial master genes seems crucial for establishing different neuronal types. SATB2 mostly works as a transcriptional repressor and *Bcl11b/Ctip2* is one of its crucial targets (Alcamo et al., 2008). In the mammalian cortex, the two genes are never co-expressed, except for two distinct neuronal subclasses projecting either to the contralateral cortex or to the brainstem, that accommodate their co-expression thanks to the action of LMO4 transcription factor (Harb et al., 2016). In fact, pallial neurons expressing only *Satb2* are exclusively present in mammals and differentiate as supra-granular pyramidal neurons. Such peculiarity seems achieved very recently in pallial evolution, as in the dorsal pallium of sauropsids *Satb2^+^* neurons always co-express *Bcl11b*, probably because of the lack of an efficient SATB2 binding region in the *Bcl11b* promoter (Nomura et al., 2018). Whether and how the existence of proper supra-granular neurons and associative pallial areas in mammals results from the differential expression of *Satb2* and *Bcl11b*, and of their target genes, in single NPCs and neurons, is still to be investigated. Not only the expression pattern of CTF genes in NPCs, but also their timing of activation seems crucial in establishing dramatic evolutionary-developmental (evo-devo) changes. Once again, the SATB2 protein played a major change in the evolution of the mammalian cortex, because its delayed heterochronic appearance in eutherians compared to metatherians may have allowed generating a novel inter-hemispheric connection, the corpus callosum (CC; Paolino et al., 2020). In any instances these and other recent observations strongly suggest that novel regulatory interactions among pallial master genes have allowed key steps in pallial evolution.

Recent evidence shows that multipotent NPCs are transcriptionally primed and already express mRNAs of genes promoting a general neuronal fate (e.g., proneural genes) or dictating a more restricted cell state, while proteins are made successively (Yang et al., 2014; Zahr et al., 2018; Telley et al., 2019); this suggests that transcriptomic studies may tell only part of the story. Therefore, for a full comprehension of how distinct cortical neuronal phenotypes are determined during development, analyses at the mRNA level should be complemented with a deeper knowledge of when and where the corresponding key proteins (and their isoforms) are made. In fact, time dependent post-transcriptional mechanisms are involved in regulating the state and potency of neuronal precursors, in addressing PMNs to the appropriate cortical layers, and in regulating several aspects of their differentiation (e.g., projection abilities, dendrite morphogenesis). This level of control relies upon several processes like: (1) mRNA alternative splicing; (2) mRNA editing; (3) mRNA stability; (4) mRNA localization; and (5) mRNA translation. The significance of these processes has been extensively reviewed elsewhere (Park et al., 2022).

In this perspective, we will provide a few key examples that underline the relevance of post-transcriptional/translational mechanisms in cortical development, focusing on RBPs, ribosomal proteins (RPs), and miRs.

### Control of cortical gene expression by RBPs and RPs

Several RBPs modulate cortical development as translational repressors. A typical example of how RBPs control the translation of specific cortical mRNAs is illustrated in the mouse by eIF4E, 4E-T, and Pum2. 4E-T and eIF4E are involved in repressing the translation of key bHLH proneural mRNAs, thus initially preventing neurogenesis in NPCs that are already primed for the process (Yang et al., 2014). In addition, a complex comprising 4E-T and Pum2 initially inhibits the translation of *Tle4* and *Brn1* mRNAs. These mRNAs are substantially co-expressed in cortical NPCs at E12 and E13, and to a lower extent at E17, but their proteins are detected only later in a mutually exclusive manner in deep or superficial cell layers respectively, in spite of the persistence of their mRNAs in these regions. Both Pum2 and 4E-T proteins can bind (directly or indirectly) the 3’UTRs of these mRNAs; while Tle4 protein appears earlier (during deep layer neurogenesis), the released translation of Brn1 protein at later stages (E16) is coincident with a declined association between *Brn1* mRNA and 4E-T in E16 RPs (during superficial layer neurogenesis; Figure 1). shRNA interference with either Pum2 or 4E-T in E13-E14 RPCs, removes the inhibition and enhances translation of the Brn1 and Tle4 proteins, causing their co-expression in both precursors and superficial neurons, and producing a deep layer Tle4-positive phenotype in superficial neurons (Zahr et al., 2018). Therefore, NPCs and derived PMNs would be primed for cell commitment and differentiation, which they fully undertake upon progressive removal of translational inhibition of key mRNAs.

Two other key RBPs involved in corticogenesis are Elavl1/HuR and Elavl4/HuD. Elavl1 plays a crucial role in the action of the translational machinery by orchestrating the repertoire of ribosomal proteins and other factors recruited in the polysomes. Interestingly, in mouse, this repertoire (“ribosome signature”) is spatio-temporally regulated and in turn influences the sets of mRNAs loaded for translation on the polysomes (Figure 1). The dynamically changing populations of mRNAs recruited for intensive translation are functionally related; these sets of mRNAs are enriched for factors involved in transcriptional and translational regulation that are different at distinct time points of neurogenesis. The dynamic “ribosome signature” is influenced by WNT3 signals, released from incoming thalamic axons, which promote *Foxp2* translation through its 3’UTR (Kraushar et al., 2014, 2015; Popovitchenko et al., 2016; Park et al., 2021).

Not only mRNAs are differentially recruited onto polysomes in a spatio-temporally dependent way, but also distinct mRNA isoforms for translational regulators are differentially engaged on polysomes from E13 to E16 in the mouse cortex. Translation of the different *Elavl4* mRNA isoforms depends upon their 5’UTR. Celf1 binds, in a time dependent fashion, these different 5’UTRs and initially prevents translation of Elavl4 isoforms (Figure 1), which subsequently dictate differentiation of glutamatergic neurons (Popovitchenko et al., 2020).

In this way, dynamic changes in the basic translational machinery are coupled to neurogenic timing and acquire an active and hitherto under-valued relevance in this process. This changing machinery influences the state of primed NPCs and PMNs so that selective translation of functionally related mRNA subsets contributes to cortical neural cell commitment and differentiation. Although it is clear that RBPs and RPs dramatically impact the developmental plan of the mammalian neocortex, little is known of their contribution to pallial evolution.

### Control of cortical gene expression by miRs

miRs inhibit the translation of target mRNAs by an imperfect pairing of their mature sequence (21–25 nt) to 3’UTR *via* interaction with the RNA interference silencing complex (RISC). Because of their nature, few miRs acting in a combinatorial way are suited for establishing robust nets of gene cross-regulation, often associated with distinct biological processes. Notably, the first 7–8 nt at the miR 5’ end—the pairing “seed” region—are crucial for the interaction with target mRNAs, thus making any single mutation critical for the repertoire of regulated mRNAs (Bartel, 2018). This peculiarity seems fundamental for the capability of miRs to rapidly co-evolve with their targets. Moreover, the structural simplicity of the hairpin-loop domain within the pre-miR precursor apparently facilitated the generation of new miR species, which arose in bursts in good correlation with key evolutionary transitions (Hertel et al., 2006; Heimberg et al., 2008; Berezikov, 2011; Londin et al., 2015; Moran et al., 2017).

A considerable number of observations indicated the pivotal role of miRs in regulating corticogenesis. In mice, pioneering studies of general inhibition of miR function by the conditional KO of DICER and RISC, responsible for miR maturation and action, demonstrated that miR-mediated RNA interference is mandatory for proper survival, expansion, and differentiation of NPCs in the neurons of the six layers (reviewed in Cremisi, 2013). Many examples of several miRs regulating cortical NPC division, identity, differentiation of cortical neurons, and neuronal activity have since been reported (reviewed in Kosik and Nowakowski, 2018). For example, miRs with pan-neuronal gene expression, such as miR-124, miR-132, and miR-9, have specific roles in mouse cortical development and function. miR-124 regulates dopaminergic modulation in the prefrontal cortex (Kozuka et al., 2019), miR-132 affects visual plasticity (Mellios et al., 2011), miR-9 controls neuronal migration and cortical layering (Clovis et al., 2012; Shu et al., 2019). Moreover, in rodent cortices, non tissue-specific miRs such as miR-128, let-7, and miR-92a can play important roles, including neuronal proliferation and differentiation (Franzoni et al., 2015; Zhang et al., 2016), layering (Shu et al., 2019), or plasticity (Letellier et al., 2014).

miRs affecting NPC identity and proliferation are particularly interesting, as the size and type of their progeny are crucial, for instance, to establish the extension of cortical areas and the proportion of supra-and infra-granular cell layers, two features profoundly discriminating pallial diversity in mammals. The extent of proliferation and pattern of division of NPCs are controlled by the miR-17-92 cluster (Bian et al., 2013; Nowakowski et al., 2013), by miR-30e and miR-181d (Nigro et al., 2012), miR-7 (Pollock et al., 2014), miR-153 (Tsuyama et al., 2015), miR-34/449 (Fededa et al., 2016), miR-214 (Shu et al., 2017), miR-15b (Lv et al., 2014), miR-20a/b and miR-23a (Ghosh et al., 2014). More recently, the role of miR-137 and miR-122, both expressed in ferret and human outer subventricular zone (OSVZ), has been tackled (Tomasello et al., 2022). mir-137 sustains prolonged proliferation of basal progenitors in the neurogenic period generating layer III-II, by maintaining cell cycle genes active, by repressing neuronal genes, and by implementing the response to proliferative extracellular factors; mir-122 acts on post-mitotic cells by slowing both differentiation and migration pace and by promoting layer III-II neuron gene expression profile. Interestingly, these two miRs are not expressed in the lissencephalic mouse cortex. Likewise, mir-3607 is expressed in the germinal zone of the gyrencephalic cortices of the ferret and several primate species, including humans, but not in the mouse cortex. By blocking APC expression, miR-3607 enhances WNT signaling, leading to an increase of progenitors that may contribute to cortical expansion in gyrencephalic species; interestingly, expression of miR-3607 in the mouse cortex at E14.5 promotes initial aRGCs and outer VZ expansion, subsequently enhanced neurogenesis, and accelerated migration of CUX1^+^ neurons, that curiously fail to end migration and enter the marginal zone (Chinnappa et al., 2022).

### The 379/410 miR cluster (*Mirg*) and mammalian brain evolution

A crucial question is whether newly generated miRNAs really contributed to pallium evolution. The recent findings on two evolutionarily novel miRs, miR-541, and miR-409, indicate that both may be crucial for the development of eutherian-specific pallial features (Figure 1). The two miRs reside in *Mirg* (miR-containing gene), inside the *Dlk1-Dio3* locus. Following an initial retroposition of a miR-free sequence (*Rtl1*) within this locus, which happened in early therians, divergent evolution led, only in eutherians, to the rapid generation of the 379/410 miR cluster (*Mirg*), comprising about 40 miRNA genes (da Rocha et al., 2008; Edwards et al., 2008; Marty and Cavaillé, 2019).

At P2, miR-409 expression levels are six-fold enriched in mouse corticospinal neurons compared to callosal projection neurons. In gain-of-function experiments in the developing mouse neocortex, miR-409 targets the 3’UTR of LMO4 transcription factor mRNA, favoring corticospinal motor neuron vs. callosal neuron identity, both in cultured cortical cells (from E14.5 embryos) and *in-utero* electroporated cortices (at E13.5), as assessed by BCL11B and SATB2 expression. On the other hand, loss-of-function experiments, performed in cultured cortical cells, did not sort significant effects, suggesting that, besides miR-409, other players may still inhibit *Lmo4* (Diaz et al., 2020).

miR-541 was characterized in an *in vitro* stem cell model of cortical development and shown to regulate the translation of the key *Satb2* mRNA (Martins et al., 2021). It is expressed in early NPCs and PMNs, then its expression declines. The binding of AGO2 to *Satb2* mRNA (demonstrated by AGO-RIP) is stronger during early *in vitro* corticogenesis than at later stages. Consistent with this, *Satb2* mRNA translation is released *in vitro* when miR-541 expression and AGO2 binding decline. miR-catch analysis also confirmed that miR-541 binds *Satb2* 3’UTR. The inactivation of miR-541 triggers robust and anticipated SATB2 translation in mouse and human cortical cells (Martins et al., 2021). These observations may be of paramount interest for mammalian cortical evolution. In fact, the recent demonstration that SATB2 protein expression is delayed in the mouse compared to the dunnart marsupial cortex and that its anticipation in mouse leads upper neurons to project through the anterior commissure instead of the corpus callosum (CC; Paolino et al., 2020) suggests that miR-541 may be a crucial component of the molecular mechanism handling this brake.

The results on miR-409 and miR-541 suggest that the *Dlk1-Dio3* locus may harbor new surprises. *Mirg* transcripts are detected in the developing early nervous system and other body districts (Tierling et al., 2006; Han et al., 2012), but its overall function in the brain has not been conclusively assessed, though several of its miRs are involved in neural disorders (Rago et al., 2014; Shi et al., 2015; Winter, 2015; Gallego et al., 2016; Tsan et al., 2016; Marty and Cavaillé, 2019). Interestingly, an initial GO analysis suggested that *Mirg* could be involved in the developmental regulation of the placenta and brain, including key factors for CC formation (Glazov et al., 2008). This seems particularly interesting considering that both *Mirg* and CC, together with the corticospinal tract, appear only in eutherians.

**Figure 2:**
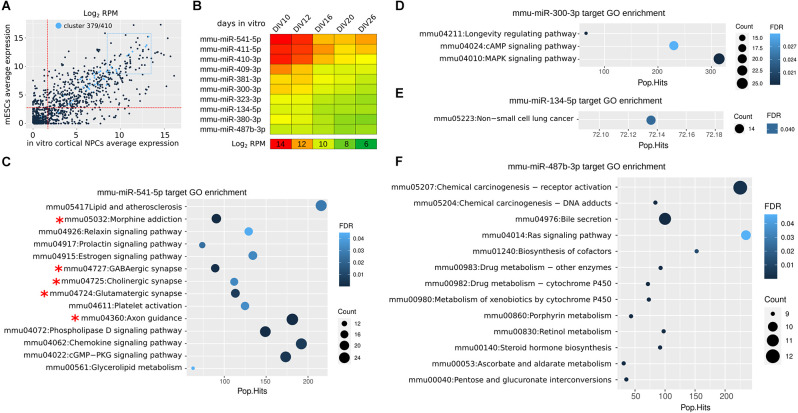
miR 397/410 cluster expression in mouse cortical NPCs. **(A)** Average expression of *Mirg* miRs in mouse NPCs (calculated from DIV10 to DIV26) as compared to expression in embryonic stem cells (mESCs). In light blue, the *Mirg* miRs. The two dotted red lines indicate the median expression levels in NPCs and mESCs; Only three out of 47 miRNAs (mmu-miR-544-3p, mmu-miR-496a-5p, and mmu-miR-299b-3p) are expressed at a lower level in both NPCs and mESCs. The dotted blue rectangle indicates the 10 more expressed miRs of *Mirg*. RPM, reads per million. **(B)** Heatmap shows the expression of the miRs from the blue dotted rectangle in **(A)**. Day *in vitro* (DIV) is the time in culture; DIV0 is the onset of the neuralization protocol. DIV10-DIV16 roughly correspond to mouse E10-E16 (Martins et al., 2021). **(C–F)** KEGG Gene Ontology (GO) enrichment of mRNAs targeted in silico by selected miRs. In silico miR-mRNA interaction was achieved by the TarPmiR tool (Ding et al., 2016) using the miRwalk online resource (http://mirwalk.umm.uni-heidelberg.de). RPM, reads per million. Pop.Hits, number of genes of the GO term. Count, the number of genes enriched in the GO term. FDR, false discovery rate. Data were obtained from Martins et al. (2021).

Cortical NPCs allow to investigate the dynamics of miR expression during *in vitro* corticogenesis (Martins et al., 2021). A new analysis indicates that the miRs of *Mirg* are highly expressed in both ESCs and cortical NPCs, as only 3 out of 47 of them are expressed less than the median level of total miRs (Figure 2A). miR-541 and miR-409 are among the most expressed in the cluster, showing a decreasing expression between day *in vitro* (DIV) 10 (roughly equivalent to mouse E10) and DIV16 (roughly equivalent to mouse E16; Figure 2B) that fits with the translational onsets of their targets *Satb2* and *Lmo4*, respectively (Diaz et al., 2020; Paolino et al., 2020). In fact, the 10 most expressed miRs of the cluster show interesting dynamics of expression during the time of *in vitro* culture (corresponding to cortical layering), suggesting their possible roles in neocorticogenesis (Figure 2B). The analysis of their in-silico targets allows to infer whether some of the miRs acquired a specific function (Figures 2C–F). Only miR-541, the most abundant in NPCs, shows targets significantly enriched in GO terms associated with neuronal features (axonogenesis and cortical synaptic activity, asterisks in Figure 2C), while the others (miR-300, miR-134, miR-487b) show enrichment in different GO terms (Figures 2D–F), or no enrichment. This observation suggests that miR-541, more than other miRs of the cluster, co-evolved with many target mRNAs involved in neural development.

### Primate specific miRs

Other novel mammalian miRs may contribute to primate pallium evolution (Kosik and Nowakowski, 2018). A key feature is the extensive self-renewal and proliferative abilities of primate oSVZ. Outer and inner sub-compartments of the macaque oSVZ show unique miR profiles, including primate-specific miRs that bind the 3’UTR of mRNAs controlling progenitor cell cycle and neurogenesis (Arcila et al., 2014). High-throughput sequencing of human NPC RNA isolated by cross-linking immunoprecipitation with AGO2 (AGO2-HITS-CLIP) allowed to define functional miRNA–mRNA modules undergoing dynamic transitions during cortical NPC maturation (Nowakowski et al., 2018). Within one of these modules, the hominid-specific miR-2115 is upregulated at the beginning of supragranular layer neurogenesis. miR-2115 resulted involved in the regulation of radial glia cell cycle by targeting ORC4, that promotes DNA replication; functional assays in both mouse developing cortex (targeting the imperfectly conserved miR-2115 site on ORC4 with a modified miR-2115) and *in vitro* cultured human radial glia (with miR-2115) lead to a lower proportion of mitotic cells and a diminution of SOX2^+^ cells (Nowakowski et al., 2018); this may favor, together with declining miR-92b action (Nowakowski et al., 2013), the transition of SOX2^+^ progenitors to TBR2^+^ cells for subsequent supragranular layer construction.

## Discussion

RBPs, RPs, and miRs play a major role in mRNA translation during corticogenesis (Figure 1).

The discovery of mammalian novel miRs targeting genes involved in the control of cell cycle, NPC identity, neuronal differentiation, and activity suggest that the rapid evolution of the neocortex might have been dramatically accelerated by novel miR/mRNA interactions. However, a better understanding of miR contribution to pallial evo-devo requires the implementation of new models of cortical development in different species, for direct analysis of miR expression and miR/mRNA interaction in the NPC progeny.

The emerging picture of neocorticogenesis figures that cells undergoing the process are, to a significant extent, initially primed for the process and already harbor key mRNAs required for the subsequent steps. The explication of this developmental pathway, therefore, relies, to a greater extent than previously assumed, on time controlled usage of these mRNAs. We here have focused on RBPs, RPs, and miRs as mere translational regulators, but mRNA usage also contemplates other intertwined key processes as alternative splicing, editing, stability, and localization, in all of which RBPs and miRs may be involved (Park et al., 2022). All these aspects, when modified, may allow new combinations of gene/protein expression that propel cortical evolution. Within the focus of this short survey, modification in RBP and miR expression certainly can produce heterochronic effects, that is, variation in the initiation, end, or duration of a process. Heterochrony may be causative of major evolutionary transitions and has been described recently for SATB2 cortical expression and for amplification of cortical progenitors (Otani et al., 2016; Nomura et al., 2018; Paolino et al., 2020; Benito-Kwiecinski et al., 2021).

## Data Availability Statement

Publicly available datasets were analyzed in this study. This data can be found here: GEO: GSE172502 (small RNA-seq) and GEO: GSE172503 (RNA-seq).

## Author Contributions

Both authors (FC and RV) equally contributed to the article and approved the submitted version.

## Funding

This work has been supported by the Open Access Publishing Fund of the Scuola Normale Superiore.

## Conflict of Interest

The authors declare that the research was conducted in the absence of any commercial or financial relationships that could be construed as a potential conflict of interest.

## Publisher’s Note

All claims expressed in this article are solely those of the authors and do not necessarily represent those of their affiliated organizations, or those of the publisher, the editors and the reviewers. Any product that may be evaluated in this article, or claim that may be made by its manufacturer, is not guaranteed or endorsed by the publisher.
